# Conduction System Pacing for Cardiac Resynchronization Therapy

**DOI:** 10.3390/jcdd10110448

**Published:** 2023-10-31

**Authors:** Óscar Cano, Javier Navarrete-Navarro, Pablo Jover, Joaquín Osca, Maite Izquierdo, Josep Navarro, Hebert D. Ayala, Luis Martínez-Dolz

**Affiliations:** 1 Electrophysiology Section, Cardiology Department, Hospital Universitari i Politècnic La Fe, Área de Enfermedades Cardiovasculares, Planta 4-Torre F. Av, Fernando Abril Martorell, 106, 46026 Valencia, Spainsaghitman@hotmail.com (H.D.A.);; 2 Centro de Investigaciones Biomédicas en RED en Enfermedades Cardiovasculares (CIBERCV), 28029 Madrid, Spain; 3 Instituto de Investigación Sanitaria La Fe, 46026 Valencia, Spain

**Keywords:** cardiac resynchronization therapy, His bundle pacing, left bundle branch pacing, conduction system pacing

## Abstract

Cardiac resynchronization therapy (CRT) via biventricular pacing (BiVP-CRT) is considered a mainstay treatment for symptomatic heart failure patients with reduced ejection fraction and wide QRS. However, up to one-third of patients receiving BiVP-CRT are considered non-responders to the therapy. Multiple strategies have been proposed to maximize the percentage of CRT responders including two new physiological pacing modalities that have emerged in recent years: His bundle pacing (HBP) and left bundle branch area pacing (LBBAP). Both pacing techniques aim at restoring the normal electrical activation of the ventricles through the native conduction system in opposition to the cell-to-cell activation of conventional right ventricular myocardial pacing. Conduction system pacing (CSP), including both HBP and LBBAP, appears to be a promising pacing modality for delivering CRT and has proven to be safe and feasible in this particular setting. This article will review the current state of the art of CSP-based CRT, its limitations, and future directions.

## 1. Introduction

**Figure 1:**
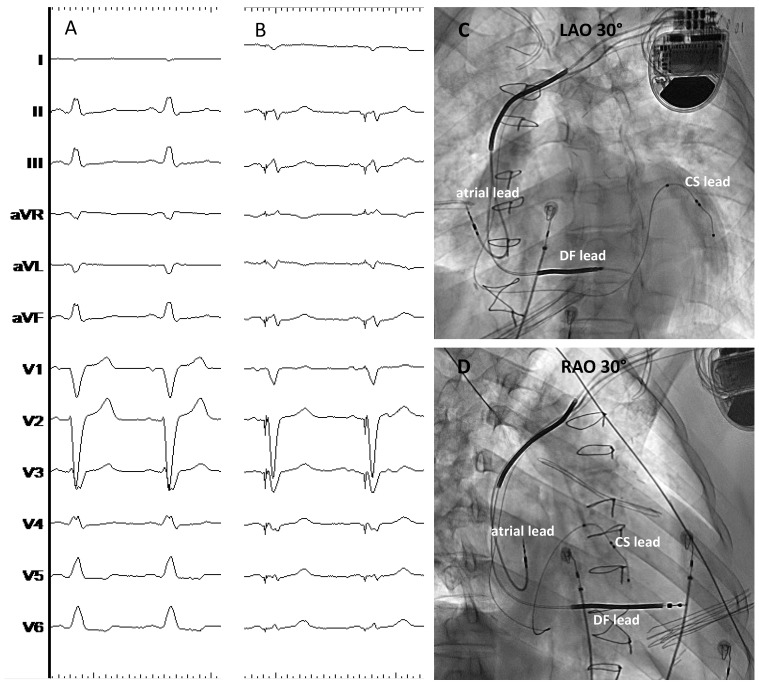
Conventional BiVP-CRT using a quadripolar CS lead in a patient with ischemic cardiomyopathy. Panel (**A**) shows the baseline QRS with LBBB; panel (**B**) shows the final paced QRS obtained with BiVP; panels (**C**,**D**) show the final lead position in the 30° LAO and RAO views, respectively. CS: Coronary sinus; DF: Defibrillation; LAO: Left anterior oblique view; RAO: Right anterior oblique view. ECG sweep speed 25 mm/s.

Cardiac resynchronization therapy (CRT) is an established treatment for patients with heart failure (HF), wide QRS, and impaired LV systolic function despite optimal medical treatment [1]. It was first described by Cazeau et al. [2] in 1994 who used four-chamber pacing (biauricular and biventricular pacing [BiVP]) for the treatment of a patient with advanced HF and a left bundle branch block (LBBB) assuming that the electromechanical dyssynchrony induced by the LBBB could be counteracted by this new pacing modality. The standard CRT technique was thereafter refined and consisted of the transvenous implantation of a right atrial lead, an RV lead, and a left ventricular (LV) lead implanted in a tributary branch of the coronary sinus (CS) in order to obtain BiVP. Since the initial description, the technique rapidly evolved and multiple observational non-randomized studies first showed significant acute hemodynamic improvements [3,4,5,6]. Subsequently, the first randomized trials demonstrated BiVP-CRT’s benefits in terms of functional capacity, peak oxygen consumption, LV ejection fraction (LVEF) improvement, and a reduction in HF hospitalizations [7,8,9,10,11,12,13]. Finally, over the next decade, multiple large randomized controlled trials showed that CRT delivered through BiVP significantly decreased mortality and HF hospitalizations [14,15,16,17,18,19,20,21,22,23,24,25]. As a result, current guidelines consider BiVP-CRT as a mainstay therapy for patients with symptomatic HF with a reduced ejection fraction and wide QRS in spite of optimal medical therapy (Figure 1).

However, approximately one-third of patients implanted with a BiVP-CRT device show no clinical or echocardiographic improvement and are considered non-responders to the therapy. Moreover, and in spite of the improvement in implant tools and device technology, there is still a small percentage of patients in which either the implant of a CS lead is not successful or, once implanted, optimal resynchronization is hampered by a high pacing threshold or by the presence of phrenic nerve stimulation [26,27,28]. As a result, different strategies have emerged in order to reduce the percentage of BiVP-CRT non-responders including the use of quadripolar LV leads and the optimization of atrio-ventricular (AV) and interventricular delay (VV) intervals, among many others [29].

Concomitantly, in recent years, a renewed interest in His bundle pacing (HBP) has emerged [30,31,32]. This physiological pacing modality aims for the restoration of the normal electrical cardiac activation sequence through the intrinsic conduction system and has been used for patients with bradycardia pacing indications [30,31,32,33,34]. Different studies have shown that HBP is able to correct intraventricular conduction disturbances including the right bundle branch block (RBBB) and LBBB. In the same manner, but more recently, LBBAP has been described as a second conduction system pacing modality (CSP) [35,36,37,38], and both techniques have been proposed as potential alternative methods for delivering CRT. This article will review the state of the art on CSP-based CRT.

## 2. Physiopathology Associated with Asynchronous LV Activation

The electromechanical dyssynchrony induced by the presence of LBBB or by conventional right ventricular (RV) myocardial pacing is the cornerstone explaining CRT’s effectiveness from a physiopathology point of view [39,40,41,42,43]. In patients with normal QRS, the myocardium is activated uniformly and the electrical waveform rapidly spreads through the His–Purkinje system and the bundle branches, resulting in a synchronized depolarization of the ventricles. The normal activation sequence includes an early transseptal activation with apex-to-base, posterior-to-anterior, and endocardial-to-epicardial electrical wave propagation. However, in the presence of an LBBB, the ventricular activation pattern changes starting in the RV as the right bundle branch function is preserved. Then, the activation waveform travels through the interventricular septum from the RV endocardium to the left ventricular (LV) endocardium, finally propagating to the endocardium of the posterolateral LV and completing a significantly slower LV ventricular activation as the electrical waveform travels through myocardial fibers not using the rapidly conducting Purkinje system.

Preclinical studies have shown that both LBBB and RV myocardial pacing are associated with poorer acute hemodynamic parameters in comparison with the normal activation observed with narrow QRS as a result of the mechanical dis-coordination leading to structural, electrical, and contractile remodeling [39,40]. At the cellular level, the dyssynchronous heart typically shows an increase in the apoptosis markers (tumor necrosis factor alpha (TNFα), caspases, and DNA fragmentation), with the development of fibrosis (increasing expression of collagen, matrix metalloproteases (MMPs), transforming growth factor beta (TGFβ), connective tissue growth factor (CTGF), and osteopontin (OPN)) and hypertrophy (increased levels of B-type natriuretic peptide (BNP), myosin heavy chain alpha (MHCα), and CTGF with a reduction in miR133) [44,45]. As a result, LBBB is associated with cardiac adverse remodeling, worsening of systolic and diastolic function, and progressive HF. BiVP plays a key role in correcting the LBBB-induced asynchrony by reducing the interventricular and intraventricular dyssynchrony.

In patients with permanent conventional RV myocardial pacing, a specific entity called “pacemaker-induced cardiomyopathy” (PICM) has also been defined to describe the detrimental effects of the asynchronous activation of the LV due to chronic RV pacing [42,43,46]. PICM has a variable incidence ranging between 10 and 30% depending on the series, and during the last 20 years, different pacing strategies aimed at physiological pacing have emerged, including algorithms to reduce unnecessary RV pacing in patients with preserved intrinsic conduction. However, these strategies are not useful in patients who need permanent RV pacing, and BiVP or CSP-based CRT could play a role in this particular scenario [47,48].

## 3. The Potential Role of CSP in CRT Candidates

HBP is a physiological pacing modality first described in 1999 by Deshmunk et al. [49]. The objective of this pacing modality is to place a pacing lead in the His bundle area in order to capture the conduction system and restore the physiological activation of the ventricles through the specific conduction system and not in a cell-to-cell fashion as with conventional myocardial RV pacing [50]. HBP was initially evaluated in patients with chronic atrial fibrillation undergoing AV node ablation and thus requiring permanent RV pacing. Subsequently, the safety and feasibility of HBP have also been demonstrated in other conduction disturbances including supra-Hisian and infra-Hisian AV block, and have also shown the capacity to correct both RBBB and LBBB in a variable percentage of patients [30,31,32,33,34]. For this reason, HBP has been proposed as an alternative or complementary technique for CRT.

More recently, a second physiological pacing modality has been described, namely left LBBAP, which includes both left bundle branch pacing (LBBP) and left ventricular septal pacing (LVSP). LBBP was first described by Huang et al. in 2017 [51], and since this initial description, observational studies have demonstrated its safety and feasibility in different scenarios including conventional bradycardia pacing indications [35,36,37,38]. Interestingly, LBBAP has been also tested in patients with wide QRS, demonstrating a high percentage of bundle branch correction with higher implantation success (85–95%) and lower complication rates when compared with HBP. Moreover, acute and mid-term electrical parameters are also superior to those previously described with HBP including lower pacing thresholds and higher R wave sensing amplitudes. As a result, LBBAP has also been investigated as an alternative or complementary technique for CRT.

Using non-invasive epicardial electrocardiographic imaging, Arnold et al. [52] identified CRT candidates in which HBP shortened the left ventricular activation time (LVAT) (18/23, 78%) and then compared the hemodynamic effects of both HBP and conventional BiVP in those patients, showing that HBP was associated with a greater reduction in QRS duration, LVAT, and the left ventricular dyssynchrony index, as well as a better hemodynamic response than conventional BiVP. In the same way, Sussenbek and colleagues recently used ultra-high-frequency electrocardiography (UHF-ECG) to compare ventricular activation patterns during BiVP and LBBAP in patients with baseline LBBB and CRT indication using two principal parameters: e-DYS (the time difference between the first and last activation in V1–V8 leads) and Vdmean (the average of V1–V8 local depolarization durations) [53]. LBBAP was associated with shorter e-DYS and shorter Vdmean than BiVP in spite of a significant reduction in the paced QRS duration in both groups, although this was greater for the LBBAP group, indicating more physiological ventricular activation with LBBAP in comparison with BiVP.

## 4. Key Concepts and Definitions for CSP-Based CRT

**Figure 2:**
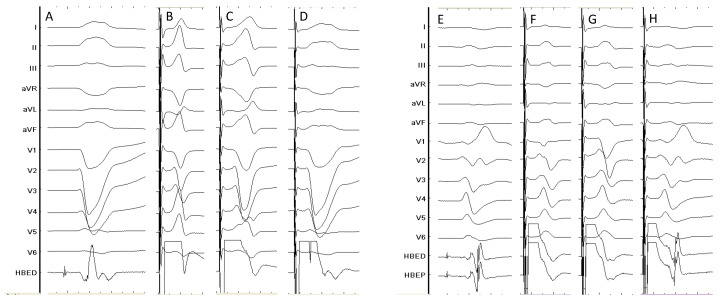
Different capture patterns during HBP-CRT. The left side of the figure shows a patient with baseline LBBB (panel (**A**)); HBP results in LBBB correction at high outputs (panel (**B**)), partial LBBB correction at intermediate output (panel (**C**)) and selective HB capture but without LBBB correction at lower output (panel (**D**)). The right side shows a patient with baseline RBBB (panel (**E**)) shows complete RBBB correction at high output (panel (**F**)), partial correction at intermediate outputs (panel (**G**)) and selective HB capture without RBBB correction at lower outputs (panel (**H**)). HBED: His bundle electrogram (distal); HBEP: His bundle electrogram (proximal). Sweep speed 100 mm/s.

HBP implies the capture of the proximal or distal His bundle resulting in a normal ventricular activation in the presence of a normal conduction through the right and left bundle branches. When no adjacent myocardium is captured, selective HB pacing is defined (S-HBP), while non-selective HBP (NS-HBP) implies the capture of both the HB and part of the surrounding myocardium [54]. Both capture patterns have been associated with comparable benefits in terms of electromechanical resynchronization. However, in patients with CRT indications, baseline wide QRS is usually present due to intraventricular conduction disturbances, typically LBBB. In this particular scenario, it is not enough to have HB capture (either selective or non-selective) but is mandatory to obtain the correction of the bundle branch block with subsequent QRS narrowing in order to be able to restore electrical synchrony (Figure 2). Thus, during HBP-CRT, up to five different capture patterns can be described including S- and NS-HBP, both with or without bundle branch correction, as well as myocardial-only capture. Every HBP-CRT capture pattern will be associated with a particular pacing threshold that should be clearly detailed in order to facilitate adequate device programming and follow-up as only bundle branch correction thresholds (either selective or non-selective) are useful to obtain cardiac resynchronization.

**Figure 3:**
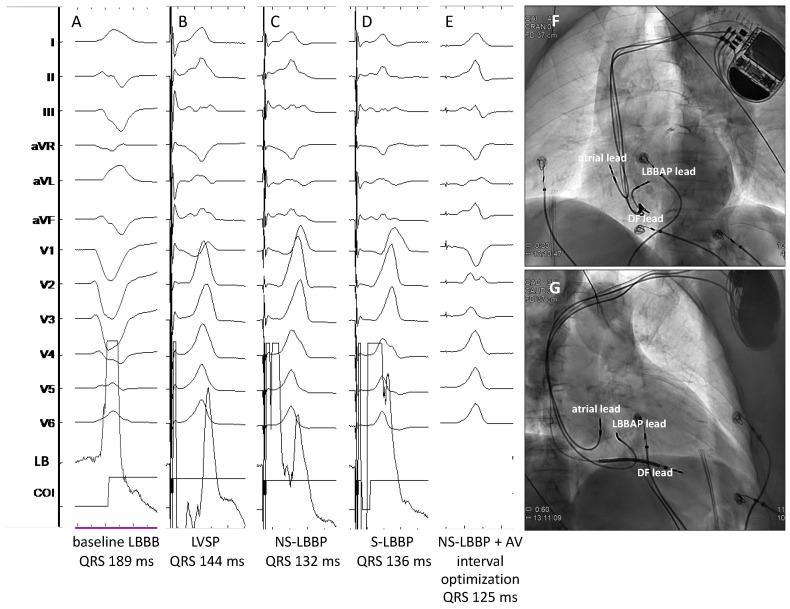
Different capture patterns during LBBAP-CRT in a patient with non-ischemic cardiomyopathy and baseline wide QRS (panel (**A**)). During the procedure, LVSP (panel (**B**)), NS-LBBP (panel (**C**)) and S-LBBP (panel (**D**)) could be observed during unipolar pacing at different outputs. Bipolar pacing with AV Interval adjusted to favor intrinsic conduction through the RBB resulted in further QRS narrowing (panel (**E**)). Panels (**F**,**G**) show the final lead position in the LAO (40°) and RAO (35°) projections, respectively. COI: Current of injury; DF: Defibrillation; LB: Left bundle; LVSP: Left ventricular septal pacing; NS-LBBP: Non-selective left bundle branch pacing; S-LBBP: Selective left bundle branch pacing. Sweep speed 100 mm/s.

On the other hand, LBBP is defined by the direct capture of the LBB or any of its fascicles together with a variable amount of the surrounding myocardium whereas LVSP is characterized by the capture of the LV septal subendocardium with subsequently rapid engagement of the left conduction system [55]. Both concepts are included under the term LBBAP and require the intraseptal implantation of a pacing lead reaching the subendocardium of the left ventricular septum. As the conduction system is captured distally to the right bundle branch during LBBAP, a delay in RV activation is typically seen with this pacing modality expressed by the characteristic r prime wave present in lead V1 (Figure 3). As both the distal and proximal dipoles of the LBBAP lead are usually within the interventricular septum, bipolar pacing may result in anodal capture, which implies that the right side of the septum is being also captured during pacing resulting in a faster activation of the RV with this particular pacing configuration and, thus, a potential benefit in terms of QRS narrowing and better electrical resynchronization. Finally, the RV activation delay induced by LBBAP-CRT can be also compensated by fusing the intact intrinsic conduction through the right bundle branch present in patients with baseline LBBB with the LBBAP wavefront adjusting the device-programmed AV interval.

## 5. Clinical Evidence of HBP-CRT

HBP is theoretically the most physiological pacing modality as it can restore the normal electrical activation pattern of the ventricles. In CRT candidates with a typical LBBB, HBP with bundle branch correction would eliminate the asynchronous activation associated with the intraventricular conduction defect. In 2013, Barba et al. [56] described the first series of 16 patients with CRT indications who underwent HBP after a failed CS lead implantation attempt. In this series, LBBB correction was temporally obtained in 81% of the cases, but permanent LBBB correction was finally achieved only in 56% due to difficulties in HBP lead fixation. The mean LBBB correction threshold at implant was high (3.09 V ± 0.44) and tended to increase at the last follow-up (3.7 V ± 0.54) with no cases of lead dislodgment. LV diameters and LVEF significantly improved during a follow-up of 31.33 ± 21.45 months. Subsequently, other mostly observational studies have evaluated the potential utility of HBP for CRT [57,58,59,60,61,62,63,64] (Table 1). Sharma et al. [58] published the largest multicenter, observational, and retrospective study of HBP in patients with different indications for CRT (primary CRT strategy, previous failed CS lead implantation, non-responders to conventional CRT) including 106 patients with a successful implant in 95 (90%). The mean BBB correction threshold was 2 ± 1.2 V at 1 ms. During a mean follow-up of 14 months, there was a significant improvement in LVEF and functional class with 6.6% of lead-related complications. In patients with a baseline LVEF < 35%, mean LVEF went from 25% at baseline to 40% at the last follow-up (*p* = 0.0001) and the NYHA functional class significantly increased from 2.8 ± 0.5 to 1.8 ± 0.6 (*p* = 0.0001). Other small, observational, single-center studies have shown similar results with significant improvement of LVEF and NYHA class [59,60].

To date, only four randomized studies have directly compared conventional BiVP-CRT with HBP-CRT [57,61,62,63]. Lutsgarten et al. [57] conducted a randomized, crossover study including 29 patients with wide QRS (>130 ms) and CRT indication who received both an LV and an HB lead and were randomized after 1 month to HBP or BiVP during 6 months and then crossover to the alternative pacing mode for 6 additional months. The HBP implant success rate was 72%, and 12 patients completed the entire protocol showing significant and comparable improvements in LVEF, NYHA class, 6-min walking test distance, and quality of life (QoL) between HBP and BiVP. The His-SYNC pilot was a multicenter, prospective, randomized controlled trial comparing BiVP-CRT with HBP-CRT in patients with conventional CRT indications [61]. A total of 41 patients were enrolled in the study with 21 randomized to HBP-CRT and 20 to BiVP-CRT. In the treatment-received analysis, patients who received HBP-CRT showed a significantly greater QRS narrowing in comparison to BiVP-CRT (125 ± 22 ms vs. 164 ± 25 ms, *p* = 0.001). After a mean follow-up duration of 12.2 months, the echocardiographic response, defined by an LVEF improvement ≥5%, tended to be higher with HBP-CRT but did not reach statistical significance. Of note, up to 48% of patients allocated to HBP-CRT crossed over to BiVP-CRT while 26% of patients initially randomized to BiVP-CRT were finally implanted with HBP-CRT. The presence of non-specific intraventricular conduction disturbance (IVCD) was the principal reason for crossover from HBP-CRT to BiVP-CRT.

In the His-Alternative trial, Vinther et al. [62] randomized 50 patients with symptomatic HF, LVEF ≤ 35%, and LBBB according to Strauss criteria to HBP-CRT or BiVP-CRT in a 1:1 ratio and were followed for 6 months. LBBB correction was achieved in up to 72% of patients in the HBP-CRT group at implant. In the per-protocol analysis, there were no differences in the LVEF improvement at 6 months between the 2 groups and HBP thresholds were significantly higher than CS lead thresholds both at implant and at follow-up. However, 7 patients crossed over from the HBP-CRT group to the BiVP-CRT group at implant while only 1 patient crossed over from BiVP-CRT to HBP-CRT. In the treatment-received analysis, LVEF was significantly higher (48 ± 8% vs. 42 ± 8%, *p* < 0.05) and the LV end-systolic volume (LVESV) was lower (65 ± 22 mL vs. 83 ± 27 mL, *p* < 0.05) in the HBP-CRT group in comparison with the BiVP-CRT group.

HBP-CRT has been also compared to BiV-CRT in patients with atrial fibrillation and LVEF < 40% undergoing AV node ablation [63]. Using a crossover design, patients received both a CS lead and an HBP lead and were randomized to either HBP-CRT or BiVP-CRT during the first 9 months and then switched to the alternative pacing mode for another 9 months. Fifty patients were enrolled but only thirty-eight patients completed the two phases of the study and were included in the final analysis. HBP-CRT was associated with a significant improvement in LVEF in comparison to BiVP-CRT. In both groups, LVEDD, NYHA class, and B-type natriuretic peptide levels significantly improved.

In summary, HBP has been evaluated in lieu of CRT in small, observational, and mainly single-center studies with limited follow-up data. To date, only 125 patients have been allocated to HBP in randomized controlled trials and compared to BiVP in patients with conventional CRT indications. Two principal concerns arise when observing the currently published data in this particular setting. The first one is that the BBB correction rate with HBP is limited and highly variable, ranging from 52 to 93% in patients included in randomized studies with baseline wide QRS. The second one is that this HBP-BBB correction rate is achieved with high pacing thresholds and a relatively high incidence of lead-related complications (up to 10.3%) including the loss of HB capture or a significant increase in the BBB correction threshold during follow-up. Finally, it should be taken into consideration that all these data come from highly specialized centers with extensive experience in CSP, so the replication of these results may not be possible in other centers.

## 6. Clinical Evidence of LBBAP-CRT

**Figure 4:**
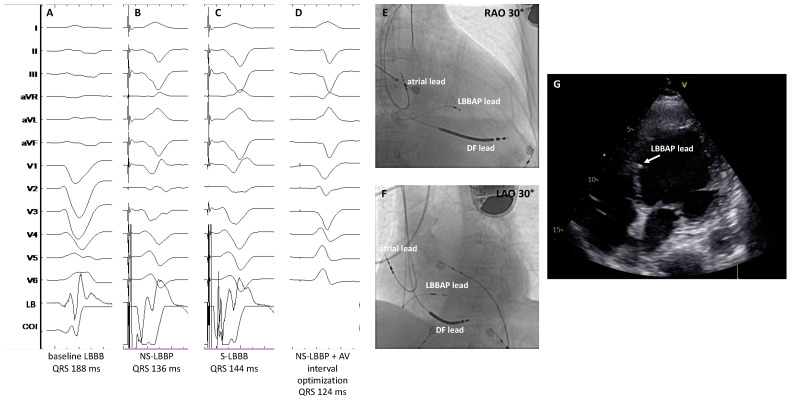
Patient with dilated cardiomyopathy undergoing LBBAP-CRT. Panel (**A**) shows the baseline QRS (188 ms); panel (**B**) shows NS-LBBP and panel (**C**) shows S-LBBP; panel (**D**) shows the final paced QRS after adjusting the programmed AV delay in the device to allow intrinsic conduction through the intact patient’s RBB resulting in further QRS narrowing; panels (**E**,**F**) show the RAO and LAO 30° view of the final lead location; panel (**G**) shows a four-chamber echocardiographic view with the LBBAP lead tip in the subendocardium of the left ventricular septum. COI: Current of injury; DF: Defibrillation; LAO: Left anterior oblique view; LB: Left bundle; LBBAP: Left bundle branch area pacing; RAO: Right anterior oblique view; RBB: Right bundle branch. Sweep speed 100 mm/s.

The first description of LBBP by Huang et al. in 2017 was in a patient with dilated cardiomyopathy, HF, and LBBB in which both CS lead implantation and HBP lead implantation failed [51]. Posterior development of the technique, with the addition of left LVSP under the term LBBAP, revealed that this new physiological pacing modality appeared to be technically easier than HBP, with higher implant success rates and was associated with lower pacing thresholds at implant and during follow-up. Thus, taking into account these findings, LBBAP was considered a potential alternative for CRT (Figure 4).

Li and colleagues published the first multicenter observational study evaluating LBBAP as a primary or rescue strategy after failed CS lead implantation in patients with conventional indications for CRT [65] (Table 2). They attempted LBBAP in 37 patients with successful implantation in 30, including 3 patients who received both an LBBAP lead and a CS lead, and compared the outcomes with 54 matched controls retrospectively recruited who had been previously treated with conventional BiVP-CRT. LBBAP-CRT resulted in significantly narrower paced QRS, a greater increase in LVEF, and greater echocardiographic response and super-response in comparison with conventional BiVP-CRT. A larger observational and retrospective series was published by Vijayaraman et al. [66] in 2021, including 325 patients with conventional indications for CRT who underwent LBBAP showing similar results: An implant success rate of 85%, optimal and stable electrical parameters, a significant reduction in paced QRS duration, and significant improvement of LVEF and NYHA class during a mean follow-up of 6 months. Other studies have consistently shown similar data in terms of a significant reduction in the paced QRS duration, optimal and stable electrical parameters during follow-up, and low lead-related complication rates associated with LBBAP-CRT [67,68].

The first multicenter, randomized controlled study comparing LBBAP-CRT with conventional BiV-CRT was published in 2022 by Wang et al. [69] A total of 40 patients with non-ischemic cardiomyopathy, LVEF ≤ 35%, and LBBB were randomized in a 1:1 fashion to LBBAP-CRT or BiVP-CRT. Two patients crossed over from LBBAP-CRT to BiVP-CRT whereas four patients randomized to BiVP-CRT finally underwent LBBAP-CRT. In the intention to treat analysis and after a follow-up of 6 months, LBBAP-CRT resulted in higher LVEF improvement, greater LVESV reduction, and greater reduction in NT-proBNP levels when compared with BiVP-CRT. However, rates of CRT response, paced QRS duration, changes in NYHA class, and 6-min walking test distance were comparable between LBBAP-CRT and BiVP-CRT. In the LEVEL-AT trial [70], 70 patients were randomized to BiVP-CRT (n = 35) or CSP-CRT (n = 35, 4 patients to HBP and 31 to LBBAP) showing a similar decrease in LVAT, LVESV, and similar rates of mortality and HF hospitalization at 6 months follow-up between the two groups in the intention-to-treat analysis.

Data on clinical outcomes comparing BiVP and LBBAP have begun to arise during the last year, principally from observational, non-randomized studies but constantly pointing towards a significant reduction in HF hospitalization with LBBAP-CRT when compared with BiVP-CRT, with no differences in overall mortality [71,72,73,74]. The largest multicenter, observational, and retrospective study published so far comparing LBBAP-CRT with BiVP-CRT included 1778 patients, 797 receiving LBBAP-CRT and 981 BiVP-CRT and provided data on clinical outcomes [74]. During a mean follow-up of 33 ± 16 months, both LBBAP-CRT and BiVP-CRT were associated with a significant increase in LVEF, but LBBAP-CRT showed a greater change in LVEF from baseline than BiVP-CRT (+13 ± 12% vs. +10 ± 12%, *p* < 0.001). The primary outcome of the study was a combined endpoint of time to death from any cause or the first episode of HF hospitalization and was significantly reduced with LBBAP-CRT compared to BiVP-CRT (20.8% vs. 28%; HR: 1.495; 95% CI: 1.213–1.842; *p* < 0.001). Secondary outcomes showed that mortality was comparable between the two groups but there was a significant reduction in HF hospitalizations in the LBBAP-CRT group (HR: 1.494; 95% CI: 1.159–1.927; *p* = 0.002).

In summary, both HBP-CRT and LBBAP-CRT are currently available techniques for delivering CRT and have been demonstrated to be safe and feasible. When directly compared to BiVP and HBP-CRT, LBBAP-CRT appears to be technically easier, with better electrical parameters and a low rate of lead-related complications [75,76,77] (Table 3). LBBAP-CRT and HBP-CRT are associated with a better acute hemodynamic response and a significantly greater improvement in LVEF than BiVP during follow-up when compared to BiVP-CRT. However, these direct comparisons arise from observational studies and should be taken cautiously. Data from randomized controlled trials are still required to draw definitive conclusions.

## 7. Combination of CSP with CS Lead Pacing-CRT

**Figure 5:**
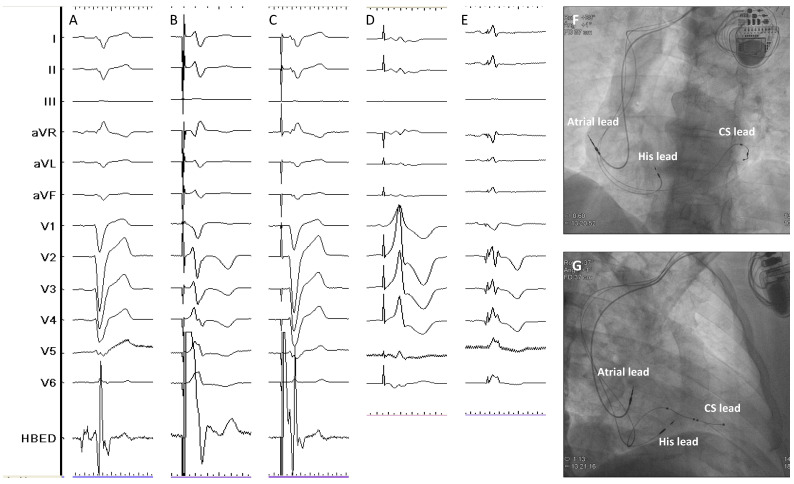
Patient with non-ischemic cardiomyopathy undergoing HOT-CRT. Baseline LBBB (panel (**A**)) could be only partially corrected with HBP (panel (**B**)). S-HBP without bundle branch correction could be seen at low outputs (panel (**C**)). Adding a CS lead and pacing from the His lead 20 ms earlier than from the CS lead, a further reduction in QRS duration could be obtained (panel (**E**)). Panel (**D**) shows the paced QRS morphology from the CS lead only. Panels (**F**,**G**) show the final lead locations in the LAO and RAO views, respectively. CS: Coronary sinus; HBED: His bundle electrogram (distal). HOT-CRT: His-Optimized cardiac resynchronization therapy. Sweep speed 25 mm/s.

There is a subset of patients in which CSP is not able to completely correct the baseline abnormal electrical activation of the ventricles. This can be explained by the presence of normal His–Purkinje activation even in the presence of a wide QRS, which reflects a primary myocardial disease and not an electrical disease. Upadhyay et al. [78] showed that among patients with LBBB patterns according to current guidelines [79], intact Purkinje activation was present in up to 36% of patients and no QRS narrowing could be obtained in this subset of patients even with demonstrated HB capture. In this scenario, and when CSP in patients with baseline wide QRS is not able to obtain a significant QRS narrowing, the combination of a CS lead with either HBP (His-optimized cardiac resynchronization therapy [HOT-CRT] or LBBAP (left bundle branch-optimized cardiac resynchronization therapy [LOT-CRT] may have beneficial effects in terms of electrical resynchronization [80,81,82,83,84] (Figure 5).

Vijayaraman et al. [80] attempted HOT-CRT in 27 patients with CRT indication and different baseline conduction disease (LBBB in 17, intraventricular conduction defect in 5, and RV pacing in 5 patients) in an observational, multicenter, and retrospective study. HOT-CRT was successful in 93% and the paced QRS was further reduced with HOT-CRT (120 ± 16 ms) in comparison with BiVP (162 ± 17 ms) or HBP alone (151 ± 24 ms), *p* < 0.0001. Moreover, LVEF and NYHA class significantly improved during a mean follow-up of 14 ± 10 months with clinical and echocardiographic responses obtained at 84% and 92%, respectively.

LOT-CRT has been also evaluated in 112 CRT candidates in another observational study reporting an implant success rate of 81% [83]. LOT-CRT resulted in a significantly greater reduction in QRS duration (144 ± 22 ms) when compared with BiVP-CRT (170 ± 30 ms) and LBBAP-CRT (162 ± 23 ms), *p* < 0.0001. With a mean follow-up of 7.8 ± 2.3 months, there was a significant improvement in LVEF and a significant reduction in NT-proBNP levels. An echocardiographic response was obtained in 62.8% and a clinical response in 76% of patients.

Results from currently ongoing randomized controlled trials such as the HIS–Purkinje Conduction System Pacing Optimized Trial of Cardiac Resynchronization Therapy (HOT-CRT) (NCT04561778) or the Conduction System Pacing Optimized Therapy (CSPOT) study (NCT04905290) are expected to shed additional light on the potential utility of both HOT and LOT-CRT.

## 8. CSP-Based CRT in Other Clinical Scenarios

### 8.1. CSP-Based CRT in Patients with Non-LBBB

Current guidelines recommend CRT for patients with symptomatic HF in spite of optimal medical treatment, LVEF ≤ 35%, and non-LBBB morphology wide QRS with a lesser degree of recommendation with respect to patients with baseline LBBB (IIa if QRS ≥ 150 ms or IIb if QRS 130–149 ms according to the ESC Guidelines [1]). However, in the MADIT-CRT trial [85], no clinical benefit was observed in patients with non-LBBB (RBBB or intraventricular conduction disturbance (IVCD)), and the echocardiographic improvements were significantly higher in patients with LBBB. The prevalence of RBBB among HF patients has been estimated at around 6.1% with a non-negligible 1-year all-cause mortality rate of 11.9%, so there is still a significant number of HF patients with non-LBBB who could be potential targets for pacing therapy according to guidelines, but with limited support in terms of clinical benefit from currently published data [86].

HBP-CRT has been evaluated in patients with baseline RBBB and CRT indications in a multicenter observational study including 39 patients (implant success rate 95%) showing acceptable bundle branch correction pacing thresholds (1.4 ± 0.7 V at 1 ms), a significant QRS narrowing (from 158 ± 24 ms to 127 ± 17 ms, *p* = 0.0001), and a significant improvement in LVEF (from 31 ± 10% to 39 ± 13%, *p* = 0.004) and NYHA class (from 2.8 ± 0.6 to 2 ± 0.7, *p* = 0.0001) during a mean follow-up of 15 ± 23 months [87]. The utility of LBBAP-CRT has also been tested in an observational study including 121 patients with standard CRT indications and RBBB [85]. The implant success rate was 88%, and LBBAP-CRT resulted in a significant narrowing of the QRS (from 150 ± 20 ms at baseline to 150 ± 24 ms, *p* = 0.01) and a significant LVEF improvement (from 35 ± 9% to 43 ± 12, *p* < 0.01). Clinical and echocardiographic response was seen in 60% and 61%, respectively. Females and those patients with a greater reduction in QRS duration with pacing (≥10 ms) obtained the maximum benefit from LBBAP-CRT in this particular setting.

In contradistinction to HBP, QRS duration reduction with LBBAP in the presence of a RBBB is challenging as the activation of the left conduction system inevitably induces a delay in RV activation so most of the QRS duration narrowing observed during LBBAP in patients with baseline RBBB is due to the septal myocardial capture obtained during non-selective LBBAP. Using a bipolar pacing configuration, anodal capture, which implies simultaneous capture from the distal and proximal poles of the pacing lead tip both located within the interventricular septum, may enhance RV septal myocardial capture and, thus, reduce RV delayed activation. However, anodal capture thresholds are usually high (>3 V in 52% of patients in the Vijayaraman et al. [88] series) so cannot be used systematically in order to reduce QRS duration in patients with RBBB undergoing LBBAP-CRT.

### 8.2. CSP-Based CRT in Patients with HF Undergoing AV Node Ablation

Patients with atrial fibrillation, HF, and impaired LVEF are candidates for AV node ablation and CRT [63,89,90,91,92]. In this setting, CSP-based CRT is a new available pacing modality. In the ALTERNATIVE-AF [63], HBP-CRT showed a significant improvement in LVEF in comparison with BiVP with similar benefits in terms of NYHA class and BNP levels between both pacing modalities.

BiVP, HBP, and LBBAP have been latterly compared in an observational, retrospective study including 50 patients with refractory AF, symptomatic HF, impaired LVEF, and narrow QRS who underwent AV node ablation and implantation of a pacing device [92]. HBP (n = 25) and LBBAP (n = 10) were associated with a significant improvement in NYHA class and LVEF whereas no significant change in both parameters was registered with BiVP (n = 13). Moreover, Rijks and colleagues have recently demonstrated that performing LBBAP and AV node ablation in the same procedure is safe and feasible [93].

### 8.3. CSP-Based CRT in Coronary Venous Lead Failure or Non-Responders to BiVP-CRT

Both HBP and LBBAP-CRT have been shown to be suitable and effective alternatives for patients with CRT indications and previous CS lead failure implants. But one step forward is to consider the potential utility of CSP-based CRT for conventional BiVP-CRT non-responders. In a multicenter, observational study, Vijayaraman et al. [94] included 44 non-responders to previous BiVP-CRT patients who underwent LBBAP or LOT-CRT by adding a pacing lead in the LBB area. LVEF and volumes significantly improved with LBBAP/LOT-CRT. In this unfavorable scenario, LBBAP/LOT-CRT was able to obtain an echocardiographic response in 40%, a super-response in 9%, and a clinical response in 45% of these previously non-responder patients. The utility of this strategy to potentially increase the CRT response and the evaluation of the risks associated with an added intervention should be tested in large randomized controlled trials.

## 9. Current Recommendations and Future Directions

CSP-based CRT is currently a promising alternative for patients with CRT indications and failed CS lead implantation that has been demonstrated to be safe and feasible. Although HBP-CRT is theoretically the most physiological pacing modality, the difficulties in lead fixation, unreliable lead stability, the limited rate of bundle branch correction, and frequently high pacing thresholds are currently hindering the spread of the use of this physiological pacing modality in patients with CRT indications. In contrast, LBBAP-CRT has the advantage of better lead stability, with lower pacing thresholds and a higher implant success rate compared to HBP and, in spite of introducing some amount of RV activation delay, has been consolidated as the preferred CSP modality for patients requiring CRT. There are still important evidence gaps regarding CSP-based CRT including the lack of long-term performance, safety and complications data, and significant concerns about LBBAP lead extractability in the future. The development of a better and wider range of implant tools, with improvements in lead design and batteries and the introduction of specific algorithms for CSP-based CRT by the manufacturers are also critical aspects of the evolution of this technique and will surely result in patient benefits.

Meanwhile, current guidelines have prudently introduced CSP-based CRT into their recommendations [1,95]. The ESC Guidelines only consider HBP-CRT an alternative to conventional BiVP-CRT after unsuccessful CS lead implantation or as an alternative to BiVP in patients with AF and HF undergoing AVN ablation [1]. More recently, the 2023 HRS/APHRS/LAHRS guidelines on cardiac physiologic pacing and mitigation of HF have widely introduced both HBP and LBBAP as an alternative to BiVP-CRT in multiple scenarios (Table 4) [95].

**Figure 6:**
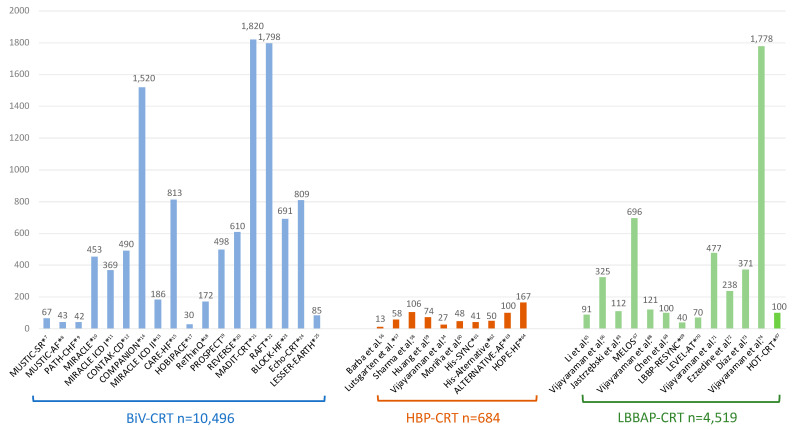
Principal studies reporting data on BiVP-CRT, HBP-CRT, and LBBAP-CRT with the total number of patients included. Asterisks indicate randomized studies [7,8,9,10,11,12,13,14,15,17,18,19,20,21,22,23,24,25,34,56,57,58,59,60,61,62,63,64,65,66,67,68,69,70,71,72,73,74,83,88,96].

Anyway, the bulk of evidence about CRT benefits still favors conventional BiVP-CRT as shown in Figure 6 with up to 10,000 patients included in randomized controlled trials reporting data on hard clinical endpoints such as mortality and HF hospitalizations. On the other hand, CSP-based CRT is a relatively new and promising technique, and data from randomized studies are still scarce but are rapidly growing, especially with LBBAP-CRT. Large multicenter observational studies are consistently showing that LBBAP-CRT is associated with a greater LVEF improvement and significantly higher reduction in HF hospitalizations in comparison to BiVP-CRT. Multiple ongoing randomized clinical trials are expected to provide more evidence in the coming years to underpin CSP-based CRT as an alternative to conventional BiVP-CRT (Table 5).

## 10. Conclusions

HBP and LBBAP are new physiologic pacing modalities that are able to provide effective CRT. Initial observational studies have shown that both techniques are safe and feasible and, in comparison to conventional BiVP-CRT, may be associated with further LVEF improvement and a significant reduction in HF hospitalizations in patients with CRT indications, but these results should be taken cautiously until randomized data become available. Ongoing randomized controlled studies should elucidate if CSP-based CRT is non-inferior or even superior to conventional BiVP-CRT.

## Figures and Tables

**Table 1:** Principal studies reporting data about HBP-CRT.

Study	Design	Patients’ Allocation	BBB Correction Rate	HBP Threshold at Implant (V) *	HBP Threshold at Follow-Up (V) *	Mean Follow-Up (Months)	Outcomes ^#^	HBP Lead Related Complications (%) ^#^
Barba et al. [56] Europace, 2013	observational, retrospective, single-centre	HBP: 16	81% temporarily 56% permanently	3.1 ± 0.4	3.7 ± 0.5	31	QRS narrowing, LVEF improvement and reduction in LVEDD and LVESD	0
Lutsgarten et al. [57] Heart Rhythm, 2015	**randomized**, crossover, multicentre	HBP: 29 BiVP: 29	72%	1.3 ± 2.2	2.4 ± 4.5	12	LVEF, NYHA class, 6MWT and QoL significantly improved with both HBP and BiVP	10.3
Sharma et al. [58] Heart Rhythm, 2018	observational, retrospective, multicentre	HBP: 106	90%	1.4 ± 0.9	2.0 ± 1.2	14	QRS narrowing, LVEF and NYHA class improvement	6.6
Huang et al. [59] Heart, 2019	observational, prospective, single-centre	HBP: 74	97% temporarily 76% permanently	1.9 ± 1.1	2.3 ± 0.9	37	QRS narrowing, LVEF and NYHA class improvement	0
Moriña-Vázquez et al. [60] Europace, 2020	observational, prospective, single-centre	HBP: 48	81%	1.6 (0.9–1.9)	0.9 (0.7–2)	6	QRS narrowing, LVEF and dyssynchrony parameters improvement	0
Upadhyay et al. [61] Heart Rhythm, 2019	**randomized**, prospective, multicentre	HBP: 21 BiVP: 20	52%	2.75 (1.3–3.4)	2 (1–3.3)	12	QRS narrowing, trend towards higher echo response with HBP vs. BiVP	0
Vinther et al. [62] JACC EP, 2021	**randomized**, prospective, single-centre	HBP: 25 BiVP: 25	72%	2.2 ± 1.2	2.4 ± 1.6	6	LVEF significantly higher and LVESV significantly lower in HBP group at 6 months	5.3
Huang et al. [63] Heart Rhythm, 2022	**randomized**, prospective, multicentre, crossover	HBP: 50 BiVP: 50	N/A, patients with baseline narrow QRS undergoing AV node ablation	0.9 ± 0.6	0.9 ± 0.6	9	significant improvement in LVEF with HBP vs. BiVP	0
Whinnet et al. [64] Eur J Heart Fail, 2023	**randomized**, crossover, multicentre	HBP: 167	93%	N/A	N/A	6	HBP did not increased peak O_2_ uptake but significantly improved QoL	5.6

* HBP threshold refers to the BBB correction threshold. Note that HB pacing thresholds were measured at different pulse widths depending on the study. ^#^ In randomized studies, outcomes and HBP lead-related complications are reported as per-protocol analyses. BBB: Bundle branch block; BiVP: Biventricular pacing; HBP: His bundle pacing; LVEDD: Left ventricular end-diastolic diameter; LVEF: Left ventricular ejection fraction; LVESD: Left ventricular end-systolic diameter; LVESV: Left ventricular end-systolic volume; NYHA: New York Heart Association; QoL: Quality of life; 6MWT: 6-min walking test.

**Table 2:** Principal studies reporting data on LBBAP-CRT.

Study	Design	Patients’ Allocation	Implant Success Rate	Pacing Threshold at Implant (V)	Pacing Threshold at Follow-Up (V)	Mean Follow-Up (Months)	Outcomes ^#^	LBBAP/CS Lead Related Complications (%) ^#^
Li et al. [65] ESC Heart Failure, 2020	observational, prospective, multicentre	LBBAP: 37 BiVP: 54	LBBAP: 81% BiVP: N/A	LBBAP: 0.81 ± 0.30BiVP: 1.22 ± 0.62	LBBAP: 0.75 ± 0.31 BiVP: 1.43 ± 0.74	6	narrower QRS, greater LVEF improvement, greater echocardiographic response and higher rate of super-responders with LBBAP vs. BiVP	LBBAP: 0 BiVP: N/A
Vijayaraman et al. [66] JACC EP, 2021	observational, retrospective, multicentre	LBBAP: 325	85%	0.6 ± 0.3	0.7 ± 0.3	6	QRS narrowing, LVEF and NYHA class improvement	2.5
Jastrzębski et al. [67] Eur Heart J, 2022	observational, retrospective, multicentre	LBBAP: 696	82%	N/A	N/A	6.4	N/A	N/A
Chen X et al. [68] Europace, 2022	observational, prospective, multicentre	LBBAP: 49 BiVP: 51	LBBAP: 98% BiVP: 91%	LBBAP: 0.92 ± 0.20 BiVP: 1.45 ± 0.39	LBBAP: 0.66 ± 0.17 BiVP: 1.42 ± 0.33	12	narrower QRS, greater LVEF improvement and higher rate of super-responders with LBBAP vs. BiVP	LBBAP: 0 BiVP: 1.8
Wang Y et al. [69] JACC EP, 2022	**randomized**, prospective, multicentre	LBBAP: 20 BiVP: 20	LBBAP: 90% BiVP: 80%	LBBAP: 0.69 ± 0.26 BiVP: 0.92 ± 0.40	LBBAP: 0.82 ± 0.20 BiVP: 1.12 ± 0.67	6	higher LVEF improvement and greater reduction in LVESV and NT-proBNP with LBBAP	LBBAP: 0 BiVP: 5
Pujol-López et al. [70] JACC EP, 2022	**randomized**, prospective, single-centre	LBBAP *: 35 BiVP: 35	LBBAP: 77% BiVP: 94%	LBBAP: 1.0 ± 0.4 BiVP: 1.2 ± 0.5	LBBAP: 0.8 ± 0.4 BiVP: 1.0 ± 0.3	6	similar decrease in LVAT and LVESV; similar rates of mortality and HF hospitalization	LBBAP: 0 BiVP: 5
Vijayaraman et al. [71] Heart Rhythm, 2022	observational, retrospective, multicentre	HBP: 87 LBBAP: 171 BiVP: 219	CSP: 86% BiVP: 75%	HBP: 1.1 ± 0.7 LBBAP: 0.8 ± 0.4 BiVP: 1.3 ± 0.6	HBP: 1.1 ± 0.7 LBBAP: 0.9 ± 0.5 BiVP: 1.4 ± 0.7	27	greater improvement of LVEF with CS; combined outcome of death or HF hospitalization lower with CSP vs. BiVP	HBP: 2.3 LBBAP: 0.6 BiBP: 0.5
Ezzedine et al. [72] Heart Rhythm, 2023	observational, retrospective, multicentre	HBP: 69 LBBAP: 50 BiVP: 119	N/A	HBP: 1.29 ± 1 LBBAP: 0.92 ± 0.54 BiVP: N/A	HBP: 1.46 ± 1.14 LBBAP: 0.86 ± 0.5 BiVP: N/A	9	greater proportion of CRT responders in CSP groups vs. BiVP. No differences in overall survival or time to first HF hospitalization	HBP: 11.1 LBBAP: 2.1 BiVP: 2.5
Díaz et al. [73] JACC EP, 2023	observational, prospective, multicentre	LBBAP: 128 BiVP: 243	LBBAP: 84.4% BiVP: 94.7%	N/A	N/A	11	higher LVEF improvement with LBBAP; significant reduction in all-cause mortality or HF hospitalization with LBBAP	LBBAP: 7 BiVP: 6.2
Vijayaraman et al. [74] JACC, 2023	observational, retrospective, multicentre	LBBAP: 797 BiVP: 981	N/A	LBBAP: 0.72 ± 0.4 BiVP: 1.15 ± 0.7	LBBAP: 0.74 ± 0.3 BiVP: 1.31 ± 0.7	33	higher LVEF improvement with LBBAP and higher proportion of patients with NYHA class improvement; significant reduction in time to death or HF hospitalization with LBBAP	LBAP: 1.3 BiVP: 2.5

* This study included 4 patients with HBP-CRT. ^#^ In randomized studies, pacing thresholds, outcomes, and HBP lead-related complications are reported as per-protocol analyses. BBB: Bundle branch block; BiVP: Biventricular pacing; CS: Coronary sinus; HBP: His bundle pacing; LBBAP: Left bundle branch area pacing; LVEF: Left ventricular ejection fraction; LVESV: Left ventricular end-systolic volume; NYHA: New York Heart Association.

**Table 3:** Comparison of procedural and follow-up outcomes with different CRT techniques. Estimation of the effect of the different pacing modalities has been obtained from pooled data from the references. Green has been added when both comparisons with the alternative groups are favorable; yellow shows if one of the comparisons is favorable and the other is neutral; orange shows one comparison is favorable and the other unfavorable; and red shows when both comparisons are unfavorable.

	BiVP-CRT	HBP-CRT	LBBAP-CRT	Reference
Procedural time	lower than HBP higher than LBBAP	higher than BiVP higher than LBBAP	lower than BiVP lower than HBP	[61,62,64,66,69,70,71,73,74]
Fluoroscopy time	higher than HBP higher than LBBAP	lower than BiVP comparable to LBBAP	lower than BiVP comparable to HBP	[56,60,62,63,68,69,70,71,73,74]
Acute CS/CSP lead threshold	lower than HBP higher than LBBAP	higher than BiVP higher than LBBAP	lower than BiVP lower than HBP	[56,57,58,59,60,61,62,63,65,66,68,69,70,71,72,74]
Acute haemodynamic effects	worst than HBP worst than LBBAP	better than BiVP comparable to LBBAP	better than BiVP comparable to HBP	[76]
Paced QRS duration	wider than HBP wider than LBBAP	narrower than BiVP comparable to LBBAP	narrower than BiVP comparable to HBP	[75,76,77]
Change in LVEF	lower than HBP lower than LBBAP	greater than BiVP comparable to LBBAP	greater than BiVP comparable to HBP	[75,76,77]
Follow-up CS/CSP lead threshold	lower than HBP higher than LBBAP	higher than BiVP higher than LBBAP	lower than BiVP lower than HBP	[56,57,58,59,60,61,62,63,65,66,68,69,70,71,72,74]
CS/CSP lead-related complications	lower than HBP comparable to LBBAP	higher than BiVP higher than LBBAP	comparable to BiVP lower than HBP	[56,57,58,59,60,61,62,63,64,65,66,68,69,70,71,72,73,74]

BiVP-CRT: Biventricular pacing cardiac resynchronization therapy; CS: Coronary sinus; CSP: Conduction system pacing; HBP-CRT: His bundle pacing cardiac resynchronization therapy; LBBAP-CRT: Left bundle branch area pacing cardiac resynchronization therapy; LVEF: Left ventricular ejection fraction.

**Table 4:** Current CRT recommendations from the 2021 ESC guideline on cardiac pacing and CRT and the 2023 HRS/APHRS/LAHRS guideline on cardiac physiologic pacing for the avoidance and mitigation of heart failure.

Clinical Scenarios	2021 ESC Guideline on Cardiac Pacing and CRT [1]	Clinical Scenarios	2023 HRS/APHRS/LAHRS Guideline on Cardiac Physiologic Pacing [95]
HF, SR, LVEF ≤ 35%, LBBB, QRS ≥ 150 ms	BiVP-CRT (I-A) HBP if unsuccessful CS lead implantation (IIa-B)	HF, LBBB, LVEF ≤ 30%, NYHA class I	BiVP-CRT (2b, B-R)
HF, SR, LVEF ≤ 35%, LBBB, QRS 130–149 ms	BiVP-CRT (IIa-B) HBP if unsuccessful CS lead implantation (IIa-B)	HF, LBBB, QRS ≥ 150 ms, LVEF ≤ 35%, NYHA class II-IV	BiVP-CRT (1, A) HBP or LBBAP if BiVP-CRT cannot be achieved (2a, C-LD)
HF, SR, LVEF ≤ 35%, non-LBBB, QRS ≥ 150 ms	BiVP-CRT (IIa-B) HBP if unsuccessful CS lead implantation (IIa-B)	HF, LBBB, QRS 120–149 ms, LVEF ≤ 35%, NYHA class II-IV	BiVP-CRT (1, A) if female sex BiVP-CRT (2a, B-R) for the rest
HF, SR, LVEF ≤ 35%, non-LBBB, QRS 130–149 ms	BiVP-CRT (IIb-B) HBP if unsuccessful CS lead implantation (IIa-B)	HF, LBBB, QRS ≥ 150 ms, LVEF 36–50%, NYHA class II-IV	BiVP-CRT (2b, C-LD) HBP or LBBAP (2b, C-LD)
HF, AF, LVEF ≤ 35%, LBBB, QRS ≥ 130 ms, NYHA class III-IV	BiVP-CRT (IIa-C) HBP if unsuccessful CS lead implantation (IIa-B)	HF, non-LBBB, LVEF ≤ 35%, QRS 120–149 ms, NYHA class III-IV	BiVP-CRT (2b, B-NR) HBP or LBBAP (2b, C-LD)
HF, LVEF ≤ 35%, previous PM/ICD with high VP burden	BiVP-CRT (IIa-B) HBP if unsuccessful CS lead implantation (IIa-B)	HF, non-LBBB, LVEF ≤ 35%, QRS ≥ 150 ms, NYHA class II	BiVP-CRT (2b, B-R) HBP or LBBAP (2b, C-LD)
Symptomatic AF, LVEF < 40% candidates for AVN ablation	BiVP-CRT (I-B) HBP if unsuccessful CS lead implantation (IIa-B) HBP (IIb-C)	HF, non-LBBB, LVEF ≤ 35%, QRS ≥ 150 ms, NYHA class III-IV	BiVP-CRT (2a, A) HBP or LBBAP if BiVP-CRT cannot be achieved (2b, C-LD)
Symptomatic AF, LVEF 40–49% candidates for AVN ablation	BiVP-CRT (IIa-C) HBP if unsuccessful CS lead implantation (IIa-B) HBP (IIb-C)	Pacemaker indication, LVEF 36–50% and anticipated high VP burden	BiVP-CRT (2a, B-R) HBP or LBBAP (2a, B-NR)
Symptomatic AF, LVEF ≥50% candidates for AVN ablation	BiVP-CRT (IIb-C) HBP if unsuccessful CS lead implantation (IIa-B) HBP (IIb-C)	Pacemaker indication, LVEF 36–50%, LBBB and anticipated low VP burden	BiVP-CRT (2b, C-LD) HBP or LBBAP (2b, C-LD)
SR or AF, pacing indication for high degree AV block and LVEF < 40%	BiVP-CRT (I-A) HBP if unsuccessful CS lead implantation (IIa-B)	PICM with HF and high burden RVP	BiVP-CRT (1, B-NR) HBP or LBBAP (2b, C-LD)
		AF + AVN ablation + LVEF ≤ 50%	BiVP-CRT (2a, B-R)

AF: Atrial fibrillation; AVN: Atrioventricular node; BiVP-CRT: Biventricular pacing cardiac resynchronization therapy; HBP: His bundle pacing; HF: Heart failure: SR: Sinus rhythm; LVEF: Left ventricular ejection fraction; LBBAP: Left bundle branch area pacing; LBBB: Left bundle branch block; NYHA: New York Heart Association; PICM: Pacemaker induced cardiomyopathy; RVP: Right ventricular pacing; VP: Ventricular pacing.

**Table 5:** Principal ongoing or planned randomized trials on CSP-based CRT. Reprinted with permission from Ref. [97].

Study	Comparison	Design	Inclusion Criteria	Sample	Primary Outcome	Mean FU (Months)	Secondary Outcomes
His-ALTERNATIVE-II NCT05814263	HBP/LBBAP vs. BiVP	randomized parallel	HF, LVEF ≤ 35%, QRS > 130 ms, typical LBBB	40	CRT response by change in LVESV	6	LVEF, QoL, 6MWD, NT-proBNP, QRS duration, device complications
Left Bundle CRT NCT05434962	LBBAP vs. BiVP	randomized parallel	HF, LVEF ≤ 35%, QRS > 130 ms, typical LBBB	176	CRT response by change in LVESV or clinical composite score	12	LVEF, QoL, 6MWD, death, HF hospitalization, ventricular arrhythmias, device complications
Left vs. Left NCT05650658	HBP/LBBAP vs. BiVP	randomized parallel	HF, LVEF ≤ 50%, QRS > 130 ms or anticipated VP > 40%	2136	Combined all-casue mortality or HF hospitalization	66	QoL, NYHA, 6MWD, death, CV death, hospitalization, CV hospitalization
CONSYST-CRT NCT05187611	HBP/LBBAP vs. BiVP	randomized parallel	QRS > 130 ms, LVEF < 35–40%	130	Combined all-cause mortality, cardiac transplant, HFH, LVEF improvement < 5 points	6	LVEF, LVESV, HFH, mortality, QRS duration, NYHA, correction of septal flash
PhysioSync-HF NCT05572736	HBP/LBBAP vs. BiVP	randomized parallel	HF, LVEF ≤ 35%, QRS ≥ 130 ms, typical LBBB	304	Non-inferiority of clinical benefit	12	Composite (all-cause death, HFH, urgent HF visit), cost-efectiveness, QoL, 6MWD, NYHA, NT-proBNP, QRS duration
CSP-SYNC NCT05155865	HBP/LBBAP vs. BiVP	randomized parallel	LVEF ≤ 35%, QRS ≥ 130 ms, typical LBBB, NYHA II–III	60	LV volume, LVEF, NYHA, NT-proBNP, 6MWD, QoL	6	myocardial work redistribution, QRS duration, procedure complications, arrhythmias
RECOVER-HF NCT05769036	LBBP vs. BiVP	randomized parallel	QRS > 130 ms, LVEF < 35–40%, NYHA ≥ II	60	Combined all-cause mortality or HFH	24	all-cause mortality, CV mortality, HFH, CRT-D shocks, LVEF, QoL, NYHA

## Abbreviations

AV	atrioventricular
BiVP	biventricular pacing
CS	coronary sinus
CSP	conduction system pacing
CRT	cardiac resynchronization therapy
HBP	His bundle pacing
HF	heart failure
HOT-CRT	His-optimized cardiac resynchronization therapy
IVCD	intraventricular conduction disturbance
LBB	left bundle branch
LBBAP	left bundle branch area pacing
LBBP	left bundle branch pacing
LBBB	left bundle branch block
LOT-CRT	left bundle branch-optimized cardiac resynchronization therapy
LV	left ventricle
LVAT	left ventricular activation time
LVEF	left ventricular ejection fraction
LVESV	left ventricular end-systolic volume
LVSP	left ventricular septal pacing (LVSP)
NS-HBP	non-selective HB pacing
NYHA	New York Heart Association functional class
PICM	pacemaker induced cardiomyopathy
QoL	quality of life
RBBB	right bundle branch block
RV	right ventricle
S-HBP	selective HB pacing
VV	interventricular interval
